# Heating of Leicester Infirmary

**Published:** 1915-08-21

**Authors:** 


*To the Editor of* The Hospital.

### Heating of Leicester Infirmary.

*To the Editor of* The Hospital.

Sir,—Our attention has been drawn to an article in your valued paper with reference to the heating of hospitals, and reference is given to the Leicester Infirmary.

In the first paragraph on p. 382 it is mentioned that "the heating of the wards is carried out partly on the Plenum system and partly on radiators." This is wrong so far as the "Plenum" is concerned. We wish to point out that the "Plenum" system is not installed in any portion of the hospital wards. The "Plenum" is strictly applied to the *out-patients' department* and the *large operating theatre*. We should be glad if you would please have this corrected so that there can be no misunderstanding. Apologising for troubling you on this matter. We are, yours faithfully,

Ashwell & Nesbit, Ltd.

Leicester, August 12, 1915.

⁂ We regret that the writer misunderstood the position, for he described the system as it was explained to him.—Ed. The Hospital.

